# Chronic Kidney Disease of Unknown Etiology in India: What Do We Know and Where We Need to Go

**DOI:** 10.1016/j.ekir.2021.07.031

**Published:** 2021-08-09

**Authors:** Oommen John, Balaji Gummudi, Anubhuti Jha, Natarajan Gopalakrishnan, Om P. Kalra, Prabhdeep Kaur, Vijay Kher, Vivek Kumar, Ravi Shankar Machiraju, Nicolas Osborne, Subrata Kumar Palo, Sreejith Parameswaran, Sanghamitra Pati, Narayan Prasad, Vinay Rathore, Mohan M. Rajapurkar, Manisha Sahay, Ravi Raju Tatapudi, Jarnail S. Thakur, Vidhya Venugopal, Vivekanand Jha

**Affiliations:** 1 George Institute for Global Health India, UNSW, New Delhi, India; 2 Prasanna School of Public Health, Manipal Academy of Higher Education, Manipal, India; 3 Institute of Nephrology, Madras Medical College, Chennai, India; 4 Pt BD Sharma University of Health Sciences, Rohtak, India; 5 National Institute of Epidemiology, Chennai, India; 6 Medanta Kidney & Urology Institute, Medanta the Medicity, Gurugram, India; 7 Department of Nephrology, Postgraduate Institute of Medical Education and Research, Chandigarh, India; 8 Seven Hills Hospital, Vishakhapattanam, India; 9 School of Public Health University of Queensland Herston Australia; 10 School of Population Health University of New South Wales Australia; 11 ICMR Regional Medical Research Centre, Bhubaneswar, India; 12 Jawaharlal Institute of Postgraduate Medical Education & Research, Puducherry, India; 13 Department of Nephrology, Sanjay Gandhi Postgraduate Institute of Medical Sciences, Lucknow, India; 14 All India Institute of Medical Sciences, Raipur, India; 15 Muljibhai Patel Urological Institute, Nadiad, India; 16 Department of Nephrology, Osmania General Hospital, Hyderabad, India; 17 Apollo Hospital, Vishakhapattanam, India; 18 School of Public Health, Postgraduate Institute of Medical Education and Research, Chandigarh, India; 19 Sri Ramachandra Institute of Higher Education and Research, Chennai, India; 20 School of Public Health, Imperial College, London, UK

**Keywords:** chronic kidney disease, chronic kidney disease of uncertain etiology, climate change, heat stress nephropathy

## Abstract

Chronic kidney disease (CKD) not associated with known risk factors has been reported from parts of India and is presumed to be similar to CKD of unknown etiology (CKDu) that has been described from Central America. The reports from India have been fragmented without clear description of the disease phenotype or its determinants. This paper summarizes the current state of knowledge around CKDu in India based on a review of literature, multi-stakeholder consultation, and a survey of Indian nephrologists. We also contacted individual research groups to solicit data. Our findings suggest that that CKDu is reported from most regions in India; however, it is interpreted differently from the phenotype described from Central America and Sri Lanka. The differences include lack of a clear demographic or occupation group, older age of affected participants, and presence of mild hypertension and low-grade proteinuria. Well-designed prospective field studies with appropriate diagnostic workup are needed to establish the disease burden and identify etiologies, along with socioeconomic and health consequences, the intersection with the environment, and the public health response. Community-based research should phenotype the entire CKD population rather than be restricted to cases with presumed CKDu based on predefined criteria. Guidelines are needed for clinical evaluation, referral, management, and harmonization of clinical documentation and health records. More data are needed to support the existence of a unique CKDu phenotype in India.

In the past 2 decades, a form of CKD has been described in people without any known risk factors such as diabetes, hypertension, glomerulonephritis, or genetic kidney disease from several geographically distinct, predominantly rural locations in diverse regions across the world.^1^ Initially reported from Central America^2^ and Sri Lanka,^3^ this entity has now been documented or suspected in Nicaragua, El Salvador, Costa Rica, Guatemala, Mexico, Panama, Sri Lanka, India, Egypt, Tunisia, Cameroon, Egypt, South Africa, the Philippines, Taiwan, Indonesia, Thailand, the United States, and the United Kingdom.^4^

There is no agreement on whether the CKD in all these clusters represents a single disease or a group of different diseases. The clinical features indicate the presentation to be consistent with the predominant tubulointerstitial pattern of injury. As the cause of these nephropathies is not clear, the term chronic kidney disease of unknown etiology (CKDu) has been used. Other names include CKD of nontraditional origin, Mesoamerican nephropathy, and chronic interstitial nephritis in agricultural communities.^5^ This diagnosis is primarily one of exclusion, but based on the initial reports from Central America, certain criteria have been proposed — including young age, lack of known CKD risk, and presentation with reduced glomerular filtration rate, minimal proteinuria, and no or slight increase in blood pressure.^6^

## CKDu in India

The first description resembling what would later become known as CKDu can be found in a 1993 report by Mani.^7^ He described chronic interstitial nephritis as the leading cause of chronic renal failure in patients presenting to a single hospital in Chennai. The overall prevalence of chronic interstitial nephritis in this report was 28%, increasing to 38% among lower-income patients. Approximately 70% presented with advanced kidney failure.^7^

Around the same time, cases of unexplained kidney failure were rising in a region known as Uddanam in the coastal district of Srikakulam in Andhra Pradesh, as reported by the local press.^8^ By 2015, an estimated 34,000 people had been diagnosed with CKD with about 4500 reported deaths.^9^ This condition attracted national and international attention and received the eponym Uddanam nephropathy. Soon after, nephrologists in other parts of the country started reporting similar presentations.^8^ Unfortunately, no studies were performed, and there remained a degree of confusion on what constitutes CKDu in India because it is common for patients with kidney disease due to other conditions such as diabetes, glomerulonephritis, and vascular disease to remain undiagnosed and present late.

In 2005, the CKD Registry of India Working Group suggested a diagnostic category of “CKD — cause undetermined.” This was deemed to be a diagnosis of exclusion, and no definition was provided. In the Registry report containing data on 52,273 patients, this category emerged as the second most common cause of CKD (16%) after diabetic nephropathy (31%). An additional 7% were labelled as chronic interstitial nephritis.^10^ In 2019, O’Callaghan Gordo *et al.*^11^ published the results of secondary data analysis of three population-based studies. They found a higher prevalence of CKD without diabetes, hypertension, or heavy proteinuria among rural populations in southern India. In the meantime, several research groups visited Uddanam, surveyed the local population, and tested the water for contaminants, but the findings were inconclusive and not published.

This paper outlines the current landscape of CKDu in India, its socioeconomic and health consequences, possible intersection with the environment, the public health response, and suggested a research agenda.

The analysis presented in this paper is based on

1) Literature review: using standardized methods, we identified peer-reviewed papers and public reports that examined the burden, risk factors outcomes, and policy aspects of CKD of undetermined cause in India.
2) We supplemented the literature review with
  a) A search of the grey literature and interviews with nephrologists and other experts to gain understanding around perceptions of the burden, determinants, cause and outcome of CKD where the cause is not identifiable. We also sought their opinion on the landscape around research, policy and operational aspects of CKD care.
  b) An online survey to the membership of the Indian Society of Nephrology. A total of 110 of 1687 members returned the survey. The results of the survey are tabulated in Table 1.
3) Two national stakeholder consultations — the first one on the side lines of the Annual Congress of the Indian Society of Nephrology at Chandigarh in November 2019, and an online meeting in May 2021. A total of 26 experts in CKDu research and policy response participated in two roundtable discussions.

On the basis of this review, we were unable to frame CKDu in India in the same way that it has been done in Central America — in particular, the strong anchoring to certain professions, younger age, male preponderance, and the almost complete absence of proteinuria and hypertension. Further, the diagnostic label has been used loosely for all cases where an etiologic diagnosis was not apparent. Therefore, we present the analysis of CKDu as it is understood in India, because it has a bearing on future research and priority-setting.

**Table 1:** Survey results on CKDu clinical and research practices in India

Parameter	Reponses (n = 110)
How many new cases with CKD do you see in your practice in a month?	25 ± 45
In how many do you make a presumed diagnosis of CKDu?	4 ± 6.5
What is the age of patients with CKDu?	35 ± 10
Have you noticed any geographic clustering of patients with CKDu?	
Yes	38
No	45
Not sure	24
Do you maintain a record/registry of patients with CKD?	
Yes	34
No	76
Are you aware on local programs to address the burden of CKD?	
Yes	31
No	79
Are you interested in participating in research on CKDu?	
Yes	88
No	2
Maybe	20

CKD, chronic kidney disease; CKDu, chronic kidney disease of unknown etiology.

## CKDu in Different Locations of India

### Andhra Pradesh and Telangana

Despite the long-standing interest,^12^ findings from population-based surveys that examined the prevalence of CKD in Uddanam were published only recently. In a sample of 2210 adult participants, Tatapudi *et al.*^13^ found the prevalence of CKD at 18.3%, with 13% meeting the case definition of CKDu. This study was limited by a single time point estimate of the kidney function abnormalities and the use of unconventional definitions. The Study to Test and Operationalize Preventive Approaches for CKD of Undetermined Etiology (STOP-CKDu) study recruited participants from 67 villages in the region^14^ and found the age-adjusted CKD prevalence to be 21.3% and 16.2% among males and females, respectively. This study used a more robust methodology, including confirming all abnormal values by repeat testing. Those with estimated glomerular filtration rate (eGFR) <60 ml/min per 1.73 m^2^ were older, more likely to be uneducated, manual laborers/farmers, tobacco users, and had hypertension, heart disease and family history of CKD. Interestingly, both of these studies found the median population age to be 44 years old and showed increasing CKD risk with age. The STOP-CKDu study also found a high (42%) prevalence of hypertension (33% recent/new onset) and proteinuria (protein creatinine ratio [g/g] > 0.5 in 7% and 0.15–0.5 in 8.4%).^15^

Clusters with high CKD burden have also been reported from other districts in Andhra Pradesh, in particular, Nalagonda, Prakasam, and Nellore, suggesting this problem is likely more widespread than originally assumed.^16^, ^17^, ^18^ The STOP-CKDu investigators are following up their cohort to estimate the incidence of CKD as well as the rate and risk factors of disease progression. Biobanked samples will allow exploration of additional hypotheses. Ancillary studies are exploring heat stress with links to acute kidney injury and water quality.^19^

Gowrishankar *et al.*^20^ biopsied four patients with serum creatinine values ranging from 1.4 to 2.6 mg/dl. The findings were dominated by tubular atrophy, interstitial fibrosis, and varying degrees of ischemic changes and glomerular obsolescence.

### Tamil Nadu and Puducherry

Referral patterns in major hospitals in Chennai and Puducherry have shown a progressive increase in the number of patients presenting to the renal services with a short history, advanced kidney failure, no history of hypertension or proteinuria, and contracted kidneys. The affected population is predominantly rural and consists of male agricultural workers and those working in foundries, brick kilns, *et cetera*. Another distinct group consists of patients has a history of having recently returned from the Gulf region where they had been engaged in manual labor. These individuals had received a good bill of health at the time of employment, but were later discovered to have CKD and repatriated back (N. Gopalakrishnan, personal communication, April 9, 2021).

A recent report from a referral center at Puducherry reported on 2424 consecutive CKD patients — a disproportionate proportion (56%) of whom came from the districts of Villupuram and Cuddalore in Tamil Nadu. Approximately 52% were classified as CKDu. A subsequent community-based CKD screening program in those villages revealed a CKD prevalence of 19%.^21^

### Chhattisgarh

In recent years, reports in the lay press have highlighted CKDu in the Mahasamund and Gariaband districts. A total of 130 people have reportedly died of CKD without receiving proper medical care just in one village with a population of approximately 1800.^22^ A recent paper described a series of 12 cases evaluated in referral centers. All patients came from poor agricultural communities, and the clinical presentation was considered to be consistent with the CKDu phenotype. Kidney biopsy conducted in two cases showed extensive glomerulosclerosis, interstitial fibrosis, tubular atrophy, chronic vasculopathy, periglomerular fibrosis, and ischemic changes. Many patients regularly used over-the-counter pain killers (possibly nonsteroidal anti-inflammatory drugs) and indigenous medications (potentially containing aristolochic acid, heavy metals, or other nephrotoxins) and consumed locally produced potentially adulterated (with methanol) alcohol. A urinary toxicological screen identified high levels of fluoride in 10 and chromium in five patients.^23^

### Goa

Goa’s lay and medical community has been aware of the existence of a high prevalence of kidney disease without known risk factors in the Canacona block (population: 12,500). In a medical camp conducted in 2009, 13% of 298 screened participants had reduced eGFR, and urinary abnormalities were noted in 24%.^24^ No data were provided on risk factor prevalence. An environmental and biological audit in 2005 attributed the disease to mycotoxin in moldy cereals and food products and aromatic compounds in drinking water.^25^ An environmental study reported trace quantities of silica in groundwater, high blood lead levels, and widespread practice of nonsteroidal anti-inflammatory drugs consumption.^26^

### Maharashtra

Vidharbha, in the north-eastern region of Maharashtra, has recently been reporting a high burden of CKDu. There have been no studies, however. Ookalkar *et al.*^27^ described 19 patients from Yavatmal, with a mean age was 55.42 ± 10.5 years, mostly males, either farm laborers or engaged in outdoor work. Speculations have been made about the causal role of pesticide spraying and contamination of drinking water from ancient wells with basalt rocks.^20^

### Odisha

A high burden of CKD has been reported from several districts — Cuttack, Koraput, Malkangiri, Bolangir, Nayagarh, Boudh, and Nuapada. Narasinghpur, and Badamba blocks in the Cuttack district are regarded by the local press as hotspots. In a cross-sectional study conducted in 24 villages from Narsinghpur, 14.3% of the 2978 people between 20 and 60 years old were diagnosed with CKD, 75% of whom did not have diabetes or hypertension. More than 70% belonged to lower socioeconomic groups, and 48% were engaged in agricultural activities. The prevalence of CKD was equal in the two sexes. The prevalence was 20% or more in one-third of the sampled villages, suggesting geographic clustering.^28^ This could mean either an environmental or genetic basis to the disease, with potential for higher exposure to either environmental (contaminated food, water, or air), behavioral (use of nonsteroidal anti-inflammatory drugs, occupational activities, how foods are stored) or genetic (genes in families) factors.

### Punjab

An analysis of a subpopulation of 2002 adults randomly selected from the general population, who had participated in the WHO-STEPwise approach to surveillance (STEPS) survey for noncommunicable diseases, 46.7% showed an elevated albumin-creatinine ratio. Only 2% exhibited reduced eGFR. The population was characterized by a high (48%) prevalence of hypertension. This study did not report on rural-urban comparisons or other CKD risk factors.^29^

## The Psychosocial Consequences of CKDu

There was a widespread acknowledgment of the considerable stigma and high health care expenditure in those diagnosed with CKD in rural communities. In the affected villages, people fear a diagnosis of kidney disease because it brings social ostracization, exclusion, and loss of job and matrimonial opportunities. Villagers express a sense of hopelessness and consider kidney disease to be an inevitable occurrence bound to visit every family (Rathore V., Galhotra A., personal communication, November 18, 2020). There is widespread apathy and a fatalistic attitude. The focus on livelihoods and social stigma leads to a neglect of health issues, which creates a vicious cycle of ill-health and poverty.

Desperate patients are willing to try any remedy, leading to rampant use of unproven therapies, including those from indigenous systems of medicine that could be potentially nephrotoxic themselves. There is a widespread lack of confidence in public sector facilities, mainly because of a lack of trained nephrologists, and patients prefer seeking treatment in the private sector, which is expensive.

## Risk Factors for CKDu in India

There has been much speculation but little published research on specific environmental risk factors of CKD in India. Several hypotheses have been put forward, such as high levels of silica and other heavy metals in water, prolonged dehydration, heat stress, nonsteroidal anti-inflammatory drug use, folk medicines, and high pesticide use.^19^^,^^30^ Although many of these theories have logical backgrounds and even some evidence from other areas of the world, epidemiological evidence to build a robust evidence base is still missing.

### Contamination of Drinking Water

Contamination of drinking water is the most popular hypothesis. Several agencies and research groups have tested the water, mostly focusing on heavy metals, but have not found any consistent abnormality. One study showed high silica content in the water samples collected in the Uddanam area.^30^ The hydrochemical data in another study indicated that the groundwater is less mineralized in Uddanam compared to other regions.^31^ Khandare *et al.*^18^ found increased levels of strontium and silica in the drinking water in the Uchapally district of Andhra Pradesh. None of these, however, have been consistently shown to be linked with kidney damage. Recent data have shown high levels of total dissolved solids in water samples from Uddanam that may indicate high salinity resulting from seawater incursion into freshwater aquifers and may explain the high prevalence of hypertension and/or proteinuria.^32^

### Exposure to Agrochemicals

Tatapudi *et al.*^13^ were unable to establish any significant association between pesticide use and CKDu in Uddanam. They did not use biological markers (blood or urine concentration of pesticides) and instead were dependent on self-reporting, which is prone to recall bias. On the contrary, Ghosh *et al.*^33^ found a negative correlation between organochlorine pesticides in the blood and the eGFR in the urban population of Delhi (nonagricultural community) diagnosed with CKDu. Few pesticides currently used in India and globally have been linked with CKD, with studies being difficult due to the remote exposure compared to outcomes and the multifactorial risk factors of CKD.

### Heat Stress

Recurrent heat exposure with physical exertion and inadequate hydration^34^ leading to recurrent episodes of acute kidney injury^35^^,^^36^ progressing over time to CKD (heat stress nephropathy) is accepted as the leading cause for CKDu in Central America.^37^^,^^38^ Populations in most areas from where high CKD burden is being reported in India are often engaged in manual work in hot and humid ambient conditions. An ongoing study is measuring the link between heat stress and kidney disease in Uddanam.^19^

### Air Pollution

An ecologic link between air pollution and CKD has been shown in Taiwan and the United States.^39^^,^^40^ India has 22 of 30 most polluted cities in the world.^41^ Burning of crops and plastic waste contributes to air pollution, both in rural and urban areas. Cooking stoves dependent on biomass fuels are common in rural Indian households. Air pollution has not yet received consideration as a contributor to CKD in India.

### Other Causes

In recent years, data from Taiwan have raised the intriguing possibility of the role of past infection with leptospira in the genesis of CKDu.^42^ Leptospirosis, widespread in Tamil Nadu and Kerala until about 15 years ago, has seen a marked decline in prevalence, making its role in the genesis of CKD in India unlikely.

Finally, the role of genetic factors has not been well explored. In the STOP-CKDu study, family history of kidney disease was an independent predictor of CKD. In a genome-wide association study involving 56 cases from Jharkhand labelled as having CKDu and 40 controls, Prasad *et al.*^43^ found a significant association of CKDu with single nucleotide polymorphisms related to *NLRP4* and *PRKN* genes.^43^

## Barriers to the Understanding of CKDu in India

**Figure 1:**
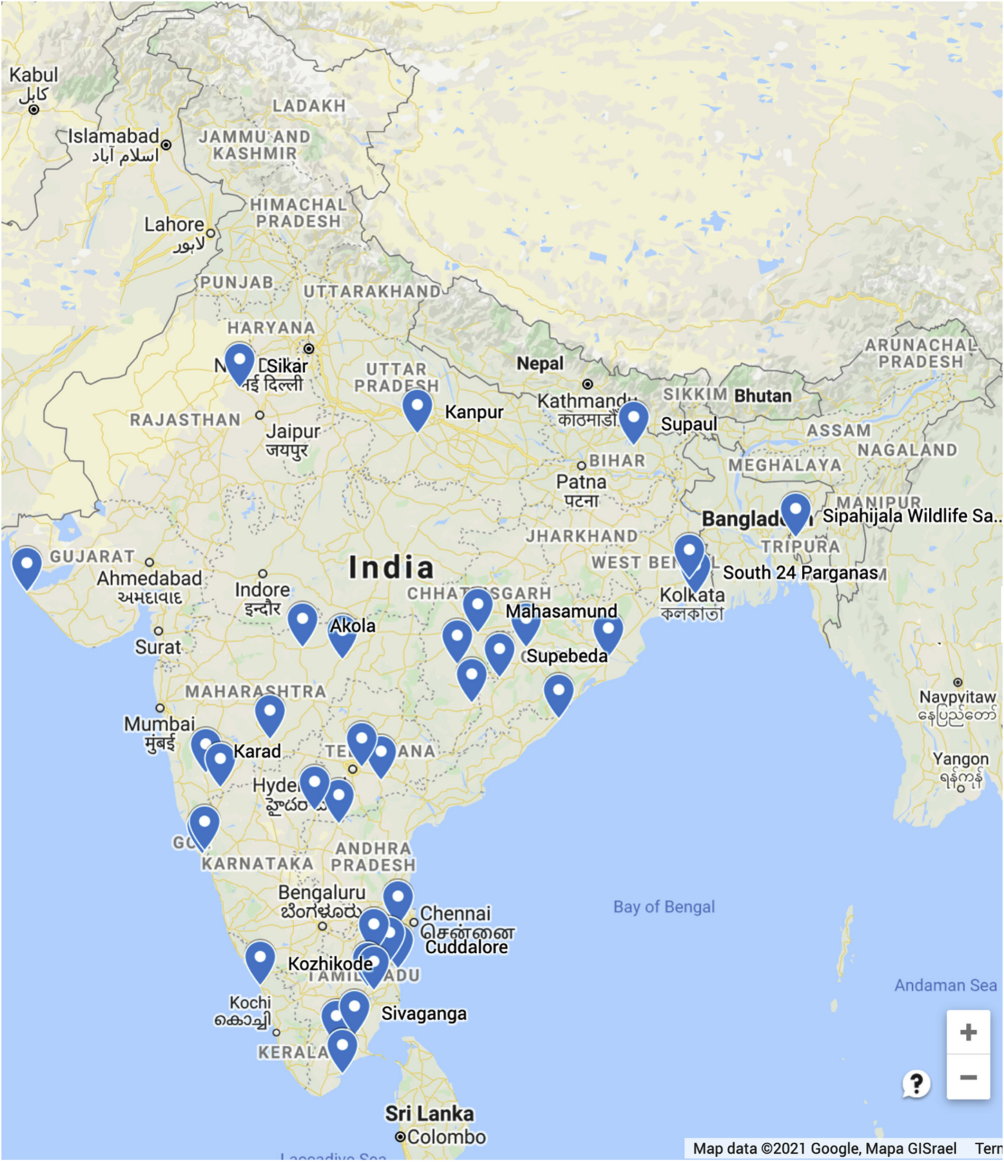
Locations from which clusters of cases with chronic kidney disease of uncertain etiology have been reported in India.

Political, cultural, socioeconomic, and health system–related factors, lack of coordinated research, and poor funding were identified as the major impediments to a full understanding of CKDu in India. Evidence suggests that CKD where the cause is unclear is more widespread beyond the clusters described thus far (Table 2, Figure 1). The piecemeal approach to characterization and phenotyping of CKD has been a handicap. For example, the Andhra Pradesh state government conducted serum creatinine testing in more than 110,000 individuals in 2017 but omitted evaluation of proteinuria or collection of data on CKD risk factors. Moreover, repeat testing or follow-up were not performed.

**Table 2:** Representation of geographic clustering of CKDu cases

State	District
Andhra Pradesh	Kadapa, Kurnool, Prakasham, Srikakulam
Chhattisgarh	Supebeda, Gariyaband, Mahasamund, Kanker, Bastar
Goa	South Goa
Gujarat	Dwaraka
Jharkhand	Saraikella
Karnataka	Raichur
Kerala	Kozhikode
Maharashtra	Osmanabad, Yavatmal, Karad, Sangli
Odisha	Balangir, Cuttack, Nayagarh, Boudh, Nuapada
Rajasthan	Sikar
Tamilnadu	Cuddalore, Villupuram, Virudhunagar, Sivaganga, Tuticorin, Ariyalur Thiruvannamalai, Perumbalur
Telangana	Nalgonda, Adilabad
Tripura	Sepahijala
Uttar Pradesh	Kanpur
West Bengal	24 Parganas, Howrah

CKDu, chronic kidney disease of unknown etiology.

Inadequate workup of cases has prevented distinction between true CKDu and those with CKD due to undiagnosed inflammatory glomerular disease and CKD accompanying metabolic, vascular, and inherited kidney diseases. The published definitions of CKDu developed for epidemiological purposes are used by clinicians to provide a quick label and to shorten patient workup. Gummidi *et al.*^15^ found widespread diagnostic and therapeutic nihilism, with most patients not undergoing even the basic diagnostic workup — only 10% of cases had even undergone renal ultrasound. There are no published kidney biopsy studies on CKDu from India.

## Implications for Population-Wide Screening for CKD in India

The government of India announced the National Programme for Prevention and Control of Cancer, Diabetes, Cardiovascular Diseases and Stroke in 2010.^44^ So far, the recommendations do not include serum creatinine and/or proteinuria testing. The increasing reports of CKD without traditional risk factors present a compelling case for population-wide screening for CKD in high burden areas. Whether such screening should be broad-based or confined to specific geographies requires more population-representative studies in the suspected high endemicity areas.

## Future Directions

We propose the following priorities for improving clinical care and research in CKD (including CKDu) in India:

1) Establishment of a multidisciplinary CKD Consortium to drive the national CKD research and response agenda — Such a group should comprise of nephrologists, epidemiologists, environmental health experts, basic scientists, social scientists, anthropologists, health economists, community groups, and patient representatives.
2) Development of guidelines for clinical and diagnostic evaluation, criteria for referral and management of CKD at all levels of health care including primary health centers and district hospitals, and dissemination of these guidelines through professional associations and teaching institutions. The recently released Standard Treatment Workflow by the Department of Health Research should be adapted.^45^ Tests for serum creatinine and proteinuria using validated techniques should be widely available.
3) Harmonization of clinical documentation and definition of a minimum dataset for ease of comparison and future research, including clinical, occupational, and family details including pedigree charting, comorbidities, medications including use of indigenous medicinal products, dietary history, hydration habits, and travel and health exposure history.
4) Community-based surveys and surveillance studies are needed. Well-designed prospective field studies will help establish the disease burden and allow complete diagnostic workups, thereby identifying etiologies. They also present opportunities for testing multiple exposures using a standard methodology.^46^ The DEGREE and CO-DEGREE consortia have proposed roadmaps for such studies. We recognize that there is no consensus on the case definition(s) for CKDu, and existing definitions^47^ are likely to be revised as new evidence emerges from ongoing studies. Therefore, a critical aim is to obtain essential information that helps in advancing the research agenda.
  a. Cohort studies or repeat cross-sectional surveys in multiple locations should be done using a standard protocol to estimate the disease prevalence, incidence and progression.
  b. Pilot projects that include serum creatinine and proteinuria assessments should be carried out as part of the National Programme for Prevention and Control of Cancer, Diabetes, Cardiovascular Diseases and Stroke in high-prevalence districts.
  c. Longitudinal studies should include systematic geospatial evaluation to ascertain clustering.
5) Case definition of CKDu:
  a. At this time there is insufficient information to define a unique CKDu phenotype in India.
  b. The prevalent CKDu case definition put forward by groups in other parts of the world should not be used to limit the diagnostic workup or clinical care.
  c. Where possible, kidney biopsies should be done and reported using standard criteria.
  d. Any community-based research should phenotype the entire CKD population rather than restricting to cases with presumed CKDu based on predefined criteria. Patients should not be excluded on the basis of predefined proteinuria cut-offs.
  e. Because an interstitial disease can also develop in those with other conditions such as diabetes and hypertension (both have a high population prevalence in India), especially in the early stages, these cases should also be included in such studies.
  f. Studies should report all cases with CKD and describe their distribution according to eGFR and proteinuria values.
6) Funding: There is a need to provide adequate funding for continued etiologic and mitigation research.
7) Community participation: Researchers should build trust with the communities where such studies are planned. Studies should be harmonized with local health systems. Individuals identified to have a health issue as part of the screening and/or research program should have access to appropriate care. Participant involvement should go beyond the standard individual informed consent and include mechanisms for sharing of results to individuals and communities. Community concerns, preferences, needs, and risk tolerance should be recognized and factored in all studies.
8) Meta-analyses, CKD observatories: Comparisons of prevalence and incidence in regions with the confirmed or suspected disease will allow systematic identification of high prevalence locations for geographic mapping.
9) Occupational health and environmental assessments should be conducted after a thorough assessment of local risk factors and include investigation of water source, food, and exposure to heat, metals, and agrochemicals using rigorous methodologies as part of multidisciplinary protocols.
10) Social and behavioral science research should proceed together to generate hypothesis regarding possible risk factors
11) Establishment of a national biorepository (including histopathology specimens) is necessary with clearly defined protocols for sample collection and data sharing for future research.
12) Implementation of a population-level program that addresses common health conditions including CKD using a community-based model of care, with appropriate escalation and referral is needed.

In conclusion, CKD is a growing health problem in India, with increasing recognition that kidney disease often develops in individuals who do not have traditional risk factors. However, there has been scant attention towards a comprehensive approach to understanding and addressing this condition. Late diagnosis and focus on the treatment of advanced kidney disease have led to the neglect of systematic evaluation of upstream factors. The link between environmental factors and CKD must be properly explored. Longitudinal studies will improve understanding of key risk factors for disease and inform policy on preventive strategies. Understanding and responding to the burden of CKDu might hold the key to attaining specific sustainable development goals (SDG) 2030 beyond the health goals (SDG 3), specifically SDG 6 that focuses on clean water and sanitation and SDG 13 that relates to climate action.

## Disclosures

VJ has research grants from Baxter, GSK and reports Consultancy and Advisory Board honoraria from Baxter Healthcare, and AstraZeneca, outside the published work. All funds were paid to his institution. VK has research grants from Novartis, Medtronics, Sanofi Aventis, Astellas India, and consultancy agreements with Torrent pharmaceuticals, Novartis, Roche, Panacea, Sanofi Aventis, Intas Pharmaceuticals, Biocon Pharmaceuticals, GSK, RPG Life Sciences, and Astra-Zeneca, India.
